# Ferroptosis and Wnt/β-Catenin Signaling Triggered by Environmentally Relevant Nanoscale Polypropylene Plastics in Human Intestinal Models

**DOI:** 10.1021/acsenvironau.5c00303

**Published:** 2026-03-18

**Authors:** Sadman Sakib, Maohui Chen, Stephanie Skerrett, Daniel Prezgot, Magda Vandenberg, Adrian F. Pegoraro, Zygmunt J. Jakubek, Zeina Maan, Shan Zou

**Affiliations:** Nanoscale Measurement, Metrology Research Centre, 6356 National Research Council of Canada, Ottawa, Ontario K1A 0R6, Canada

**Keywords:** Reference material, Nanoplastic, β-catenin, Environmental exposure, Oxidative damage, μ-IR, Atomic force microscopy

## Abstract

Nanoplastics generated through environmental weathering may disrupt epithelial barrier integrity by promoting oxidative damage and inflammation, yet most studies rely solely on pristine synthetic particles that lack surface chemistries representative of real-world aged plastics. Here, we investigated the biological effects of environmentally relevant nanoscale polypropylene reference material (NPPP-1) produced by laser ablation and evaluated its material-specific responses under matched particle-number exposure conditions, in comparison with pristine and ultraviolet-aged synthetic polypropylene in human small intestinal organoids (HSIOs) and human intestinal epithelial cells. NPPP-1 induced oxidative stress, with elevated reactive oxygen species (ROS), lipid peroxidation, and DNA oxidation, accompanied by upregulation of genes and proteins associated with inflammation, ROS, and cell death pathways. Functional assays revealed concomitant activation of ferroptosis, apoptosis, and pyroptosis. Ferroptosis was the primary driver of cell death, as evidenced by partial rescue of viability mediated by ferroptosis inhibitor ferrostatin-1. In HSIOs, NPPP-1 triggered nuclear translocation and accumulation of β-catenin and upregulated the Wnt target gene *Axin2*. Ferroptosis inhibition reduced Wnt upregulation, suggesting activation of regenerative signaling that serves to mitigate ferroptotic stress. Indeed, inhibition of this pathway increased lipid peroxidation and reduced viability, further indicating a compensatory response to counter ferroptotic stress. Imaging and spectroscopic analyses confirmed internalization of NPPP-1 within the epithelial layers, linking the presence of particles to biological effects. These findings demonstrate that environmentally relevant nanoscale plastics such as NPPP-1 elicit oxidative stress-driven cell death while simultaneously activating the Wnt/β-catenin pathway as a protective response. The combined degenerative and compensatory dynamics highlight the importance of using realistic nanoscale plastic materials and advanced 3D organoid systems to assess human health risks under conditions that mimic real-world exposures.

## Graphical abstract

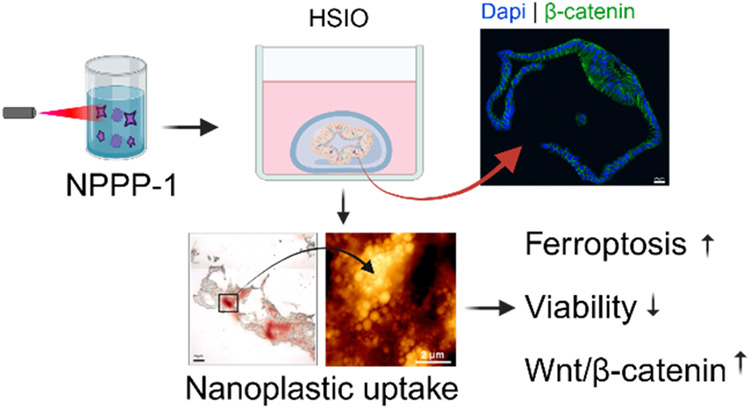

## Introduction

Nanoplastics (NPs), polymeric plastic particles with at least one dimension in the below 1 μm, and derived from degraded everyday polymer products are increasingly detected in food, water, and air; raising concerns about human exposure and epithelial injury at mucosal barriers such as the gut and lung.^1,2^ Environmental aging processes-including mechanical abrasion, ultraviolet (UV)/photo-oxidation, and biofouling-modify polymers. Compared to pristine polymers, these modified polymers demonstrate increased dispersibility, pollutant uptake and biological activity such as oxidative damage to lipids, proteins, deoxyribonucleic acid (DNA) and organelles.^3–5^ Polypropylene (PP) is one of the most commonly used plastic products and has been detected in environmental matrices, making it an important focus for the evaluation of health hazards.^6^ For example, PP along with polyethylene terephthalate (PET) has been reported to be the predominant microplastic (MP) found in human waste with 20 MP particles (50–500 μm) for every 10 g human feces, indicating accumulation, ingestion, and excretion of plastic debris by humans.^7,8^

Understanding how these plastics affect human health has been challenging. Environmental aging fundamentally changes polymer chemistry and surface properties and thus requires model systems that capture these changes. This has prompted growing efforts to understand how these transformations affect toxicity. Comparative analyses consistently show that aged plastics—those that better mimic environmental modifications—exhibit stronger biological responses than their pristine analogues across multiple end points from increased oxidative stress to reproductive and developmental defects. For example, when compared to pristine polylactic acid (PLA) MPs, UV-aged PLA MPs, which showed reduced size and increased oxygen-containing functional hydroxyl (O–H) and carbonyl (C=O) groups, produced more severe toxicity as characterized by increased oxidative DNA damage and apoptosis of germline cells in *Caenorhabditis elegans*.^9^ However, the majority of laboratory based toxicological studies rely on pristine and spherical polystyrene (PS) beads in two-dimensional (2D) monocultures, limiting the relevance to real-world exposures.^6,10,11^ To bridge this gap, we have recently developed a nanoscale plastic (with at least one dimension within 1–100 nm) reference material (NPPP-1)^12^ by employing femtosecond laser ablation to generate environmentally mimetic nanoscale plastics with nonspherical discoidal morphology spanning tens to about two hundred nanometers laterally and a few nanometer thick. These PP particles carry negative charges and environmentally relevant oxygen-containing functional groups that mimic the chemical signatures of weathered plastics.^13^ The dispersion is produced at sufficiently high particle number concentrations to support biological exposure, toxicological assessment, and methodological evaluation.

Beyond using environmental plastic mimics, it is also necessary to use biological systems that better capture the inherent complexity of living tissues. Organoid models recapitulate native cell composition and tissue architecture in a three-dimensional (3D) context.^14^ Murine and human small intestinal organoids (HSIOs) mimic gut specific epithelial morphology, stem-cell dynamics, and barrier function and thus offer a more physiologically relevant platform than 2D monocultures for studying plastic-mediated injury.^6,13^ Intestinal organoid derived epithelia have shown size specific uptake of particles and inflammatory cytokine expression after plastic exposure, validating the sensitivity of the model to gut relevant toxicants.^15^ We have also shown nanoscale plastic mediated dose dependent viability loss, increased ROS generation and inflammatory cytokine expression in murine intestinal organoids.^13^

The increase in ROS observed with NPPP-1 exposure is consistent with previous findings and reinforces its utility as an environmental mimic.^13^ Mechanistically, oxidative stress is a central axis of plastic-induced toxicity. Modified plastic particles, such as those aged in laboratory environments and in particular those that are weathered via multifactorial environmental transformations including UV radiation, mechanical abrasion, oxidation, and biofilm formations elevate ROS production, overwhelm antioxidant defenses and trigger oxidative DNA damage (e.g., 8-hydroxy-2′-deoxyguanosine (8-OHdG)), resulting in regulated cell deaths (RCD).^3,5,16–20^ Beyond reporting generic cytotoxicity responses, often characterized via loss of viability, several RCD pathways, including ferroptosis, apoptosis, pyroptosis, etc. have also been implicated. Ferroptosis is a regulated, nonapoptotic form of cell death due to lipid peroxidation modulated by increased iron accumulation and reduced antioxidative defense mechanisms. During ferroptosis mediated cell death, ROS attack the polyunsaturated fatty acids (PUFAs) in cell membranes, producing malondialdehyde (MDA) and other lipid peroxidation byproducts that compromise membrane integrity.^21–23^ Increased labile Fe^2+^ inside the cells, often detected using FerroOrange or similar probes, catalyzes these peroxidation events via the Fenton reactions.^24^ NP exposures have been shown to trigger ferroptosis^21^ in epithelial systems and in vivo respiratory models, linking NP mediated ROS to lipid peroxidation and cell death.^25^ In addition, apoptosis has been shown to be coactivated, likely due to mitochondrial damage, whereas pyroptosis, an inflammasome and Caspase 1 dependent lytic cell death, is driven by particle induced innate immune activation with NLRP3 (NOD-, LRR- and pyrin domain-containing protein 3), a cytosolic pattern-recognition receptor cited as a key sensor for plastics.^26^ Recent studies indicate that NPs can preferentially trigger ferroptosis in colon cells more than the larger MPs, reinforcing the importance of particle size and surface state.^27^ Yet, how surface-altered, environmentally aged NPs modulate ferroptosis, oxidative and inflammatory cell death pathways in epithelial systems remains largely unexplored.

Intestinal injury may not be solely degenerative, as intestinal epithelia possess intrinsic regenerative and compensatory mechanisms that can help restore tissue integrity following damage. The canonical Wnt/β-catenin pathway is the primary driver of intestinal stem cell renewal and crypt-villus homeostasis.^28,29^ The Wnt family of proteins comprises secreted glycoproteins that regulate cell proliferation, polarity, and differentiation during development and adult tissue maintenance. β-catenin is a multifunctional protein that serves as a transcriptional coactivator when stabilized in the cytoplasm. In the canonical Wnt/β-catenin pathway, binding of Wnt ligands to Frizzled (G protein coupled receptor) and LRP5/6 (low-density lipoprotein receptor-related protein 5/6) receptors inhibits the β-catenin destruction complex (composed of Axin, Adenomatous Polyposis Coli, GSK-3β (glycogen synthase kinase-3 β), and Casein Kinase 1), preventing β-catenin phosphorylation and degradation. Stable β-catenin can then accumulate and translocate to the nucleus, where it binds transcription factors to activate genes controlling stem-cell renewal.^30,31^ This pathway is also redox-sensitive and oxidative conditions can help to stabilize β-catenin via inhibition of GSK-3β which thereby enhances Wnt activity.^32–34^ Thus, oxidative microenvironments created by NPs could result in activation of Wnt signaling as a protective response. Recent works have linked plastic exposures to altered Wnt/β-catenin signaling in nematodes as well as mammalian skin, cardiac and hematopoietic stem cells.^35–38^ However, most of this evidence comes from rodent models, underscoring the need for human-relevant intestinal microenvironments that can recapitulate both injury and endogenous repair signaling.

Here, we investigated the effects of NPPP-1, an environmentally realistic nanoscale PP reference material, and compared its responses with pristine synthetic PP (SynPP) and UV-aged SynPP across multiple exposure levels in 3D HSIOs and a conventional 2D epithelial model. NPPP-1 exposure provoked substantially greater cytotoxicity than SynPP and UV-aged SynPP under matched particle-number exposure conditions. It also generated oxidative stress and lipid peroxidation and initiated ferroptosis, with concomitant apoptosis and limited pyroptosis. In 3D organoids, these injurious responses also engaged Wnt/β-catenin mediated protective activity, indicating an endogenous attempt to mitigate ferroptotic damage. These findings expand on previous observations^15^ and demonstrate the additional hazard posed by environmentally processed PP, emphasizing the need to incorporate realistic nanoscale plastic materials such as NPPP-1 materials into human health and environmental risk evaluations.

## Experimental Section

### NPPP-1 Production

Polypropylene nanoplastics (NPPP-1) were generated by cold ablation of bulk PP (PP30-SH-000155, GoodFellow). Initial Fourier transform infrared spectroscopy (FTIR) performed on this bulk PP material showed spectral features consistent with PP with no additional peaks attributable to common fillers or plasticizers within the detection limits of the technique. Laser ablation was carried out using a 1030 nm, 200 fs femtosecond laser operated at 1.4 W and 200 kHz. The beam was raster-scanned across the submerged polymer surface with 50% pulse overlap, and ablated material was continuously collected into a 500 mL recirculating Milli-Q water bath. Material removal proceeded over 72 h while translating the sample along the optical axis to expose fresh polymer to the focal volume. The resulting suspension was filtered through a 2.7 μm glass microfiber membrane to remove large fragments, and subsequently concentrated and cleaned using a 10 kDa (mPES hollow fiber, 115 cm^2^ surface area) filter via tangential flow filtration (TFF) system (Repligen KrosFlo KR2i system equipped with dual pumps and PharMed Masterflex tubing, size 16). Full fabrication and characterization details are provided in ref 13 and the certificate of analysis in ref 12.

### Cell Culture

All the cell lines were kept frozen in liquid nitrogen prior to cell seeding. Human small intestinal epithelial cells (HIEC-6, CRL-3266, ATCC) were grown in Opti-MEM I Reduced Serum Medium (31985, Gibco) supplemented with 20 mM HEPES (07299, StemCell Technologies), 10 mM GlutaMAX (35050, Gibco), 4% fetal bovine serum (FBS) (A5209502, ThermoFisher Sci.) and 10 ng/mL of Epidermal Growth Factor (EGF, 236-EG-200, R&D Systems). Caco-2 (HTB-37, ATCC) cells were cultured in Eagle’s Minimum Essential Medium (30–2003, ATCC) supplemented with 20% FBS. All the cell were used between passage 3 and 12. The cultures were carried out at 37 °C and 5% CO_2_.

### Human Small Intestinal Oranoid Culture

HSIOs, established from primary duodenal human intestinal fragments, were purchased from the Human Organoid Innovation Hub (HOIH) at University of Calgary. These organoids were grown using Human IntestiCult Organoid Growth Medium (human) (06010, StemCell Technologies) according to manufacturer’s protocol. The medium contains proprietary growth factors supporting intestinal epithelial stem cell maintenance and differentiation. Briefly, approximately 200 organoids were resuspended in 50 μL of cold Matrigel Growth Factor Reduced Basement Membrane Matrix (356231, Corning) and dispensed at the center of a well of a 24-well plate, which has been prewarmed at 37 °C for at least 2 h. After 5 min at room temperature, the plate was transferred and incubated upside down at 37 °C and 5% CO_2_ in a tissue culture incubator. The well was then topped up with 500 μL of media and cultured in a cell culture incubator. The organoids were passaged every 5–7 days. Cultures were maintained at low passage numbers (<10).

### Generation of SynPP and SynPP-UVd16

The synthesis of PP nanoparticles was adapted and scaled up from the method reported by Cassano et al.^39^ using PP pellets (Sigma-Aldrich, Cat. No. 428116–250G). Attenuated total reflection infrared spectra showed no additional peaks attributable to common additives. 60 mg (±6 mg) of 12 kDa PP pellets were dissolved in 7 mL of toluene at 125 °C for 6 h in a 50 mL round-bottom flask. Simultaneously, 15 mg of sodium cholate was dissolved in 54 mL of boiling ultrapure Milli-Q water. The aqueous solution was added to the toluene-PP mixture to form a two-phase system, which was then emulsified by vortexing at 16,000 rpm for 4 min. This was followed by probe sonication using a Cole-Parmer ultrasonic processor (130 W, 20 kHz) operated in a pulsed mode (30-s pulses alternating with 30-s rest intervals) for a total of 5 min at 40% amplitude. The resulting emulsion was immediately cooled in an ice–water bath for 6 min. This process was performed for nine separate batches, which were then combined prior to solvent removal. The total volume was reduced to approximately 90 mL using a Buchi rotary evaporator with a bath temperature of 60 °C and a rotation speed of 3; this process typically takes 4 to 6 h. The solution was filtered using 30 mm Puradisc nonsterile cellulose nitrate syringe filters with a 5 μm pore size (Cytiva Whatman, 10462500), using glass syringes, to eliminate nonemulsified or aggregated PP. Purification was conducted via tangential flow filtration (TFF), with a 10 kDa mPES filter. The system was run for 10 diafiltration volumes to remove residual sodium cholate. The final nanoparticle suspension was divided into two portions: one was retained as a nonirradiated PP (SynPP), while the other portion was exposed to UVC light for 16 days using a circular UVC reactor equipped with 16 evenly spaced bulbs to ensure uniform irradiation (SynPP-UVd16).

### Dynamic Light Scattering (DLS)

SynPP and SynPP-UVd16 were diluted by a factor of 100 (10 μL in 990 μL of Milli-Q water) for DLS measurements, using 20 and 1000 μL pipettes for accurate volume transfer. The measurements were performed using a Zetasizer Nano ZS particle size analyzer (Malvern Instruments, Worcestershire, UK). For each sample, one measurement consisting of 21 50 s-long runs was taken. The measurements were conducted in ZEN0040 disposable polystyrene cuvettes (Malvern) at 25 °C with 120 s-long initial equilibration time. Instrument settings included a fixed attenuation level of 8 and a laser position of 4.65.

Zeta potential was measured using the same instrument at 10- fold dilution in Milli-Q water without added salt. Measurements were conducted in DTS1070 disposable folded capillary cells (Malvern) at 25 °C, following a 120-s equilibration. The Smoluchowski model was applied for zeta potential calculations. Each sample was measured three times, with no delay between replicates, and each measurement consisted of 50 runs. Attenuation was automatically selected by the instrument.

### Nanoparticle Tracking Analysis (NTA)

NPPP-1, SynPP and SynPP-UVd16 were diluted 10,000-fold in Milli-Q water and analyzed using a NanoSight Pro instrument (NanoSight, Amesbury, United Kingdom) with a 405 nm laser. Samples were autoinjected at ambient temperature, and ten 1 min videos (4000 frames, 15 ms exposure) were recorded per sample. Focus was manually adjusted for consistency. Flow parameters were set to a sample flow rate of 3 μL/min, advanced speed of 50 μL/min, duration of 10s, and stabilization time of 20s.

### Exposure of 2D Cells and HSIOs to Nanoplastics

Both HSIOs and 2D cells (HIEC-6 and Caco-2) were passaged and seeded onto either 96-well plates (for viability and ROS measurements) or 12-well plates (for IHC, gene expression analysis, FerroOrange, MDA measurements) and exposed to either NPPP-1, SynPP or SynPP-UVd16. NPPP-1 was diluted in media to concentrations of 1 × 10^10^ (1.0 ± 0.1 μg/mL) and 1 × 10^11^ (9.6 ± 0.9 μg/mL) particles/mL (Table S1). For cross-literature comparability, particle-number doses were converted to approximate mass-equivalent concentrations using the certified total carbon content of the NPPP-1 stock^12^ and the measured particle number concentration (Supporting Information). SynPP and SynPP-UVd16 were diluted to 1 × 10^11^ particles/mL which correspond to ∼173 and ∼37.6 μg/mL, respectively (Supporting Information). The same volume of Milli-Q water as the highest concentration of NPPP-1 dispersion was added to media as a control. Both HSIOs and HIEC-6 cells were exposed to all the variants of NPs for 96h.

For rescue and pathway-modulation experiments, Ferrostatin-1 (FST-1, 5 μM) (SML0583–5MG, Sigma-Aldrich) was included from the onset of NPPP-1 (1 × 10^11^ particles/mL) exposure. For suppression of Wnt/ β-catenin signaling, XAV939 (10 μM, 72672, StemCell Technologies) was administered during the final 24 h of a 96 h total exposure period.

### Viability Assessment

HIEC-6 and Caco-2 cells were seeded onto 96-wells at a density of 5000 cells/well. For HSIOs, approximately 50 HSIOs were resuspended in 10 μL Matrigel and dispensed into the center of each well of a 96-well plate and allowed to gel for 30 min at 37 °C. They were then treated with NPs and small molecule inhibitors (for rescue experiments). Wells supplied with appropriate treatments but no cells or HSIOs were used as blanks. After 96 h, the media was replaced with 100 μL basal media only (Dulbecco’s Modified Eagle’s Medium/Nutrient Ham’s Mixture F-12 (36254, StemCell Technologies) for HSIOs; EMEM for Caco-2 and OptiMEM I Reduced Serum Medium for HIEC-6 cells). The CellTiter-Glo 3D Cell Viability Assay (G9683, Promega) was then used to determine the viability of as per the manufacturer’s instructions. Luminescence was recorded using a FLUOstar Omega (BMG Labtech) plate reader. Each assay was done with triplicates for each exposure conditions.

### Reactive Oxygen Species (ROS) Level Measurements

The reactive oxygen species (ROS) levels were determined using ROS-Glo H_2_O_2_ Assay (G8820, Promega). Similar to the viability assays, HIEC-6 cells (5000 cells/well) and HSIOs (50 organoids/well) were seeded into 96-well plates and treated at 1 × 10^10^ and 1 × 10^11^ particles/mL of NPPP-1 exposure. After 90h, the H_2_O_2_ substrate was added for the last 6 h of treatment. This substrate was added using serum free basal media only. The ROS assay process was then carried out as per the manufacturer’s instructions.

### Immunohistochemistry

HSIOs were seeded onto the center of a 12-well plate using a 100 μL Matrigel droplet at a density of 400 organoids/well and treated with NPPP-1. After 96h of treatment, the media was removed and the organoids were washed with ice cold phosphate buffered saline (PBS, 37350, StemCell Technologies) to dissolve the Matrigel and isolate the HSIOs. The HSIOs were then fixed with 4% paraformaldehyde (PFA, J61899.AP, ThermoFisher Scientific) and embedded in optimal cutting temperature (OCT) compound (23–730–571, FisherScientific) to generate frozen blocks. Frozen sections (10 μm) were then prepared from these tissue blocks. The sections were washed in PBS, blocked with 10% normal goat serum (50062Z, ThermoFisher Scientific) and incubated overnight at 4 °C with anti-HO-1 Rabbit mAb (82551T, Cell Signaling Technology), anti-β-catenin mAb (8814T, Cell Signaling Technology) and anti-8-OHdG (ab48508, Abcam). Fluorescence labeling was then carried out using secondary antibodies conjugated with Alexa Fluor 488 and 555. Dapi was used for labeling the nuclei. Imaging was performed on an Olympus confocal laser scanning microscope.

### MDA Assay

HIEC- 6 cells were plated in a 12-well plate at a density of 60,000 cells/well and around 400 HSIOs were cultured in each well of a 12-well plate. Both the cells and HSIOs were treated for 96 h with a vehicle control and 1 × 10^11^ particles/mL of NPPP-1. After treatment, the cells were washed three times with cold PBS. For HSIOs, the organoids were collected via washing away the Matrigel with cold PBS, then pelleted by centrifugation at 1500*g* for 5 min at 4 °C. The resulting cell and organoid pellets were lysed with 300 μL Extraction Buffer 5× (ab193970, abcam) diluted to 1× and left for 15 min on ice. The lysates were then centrifuged at 13,000*g* for 10 min at 4 °C and the supernatant was collected for assay. A malondialdehyde competitive ELISA kit (Invitrogen, EEL160) was employed to measure MDA concentration as per the manufacturer’s instructions.

### Reverse Transcription Quantitative Polymerase Chain Reaction (qRT-PCR)

HSIOs were treated in 12-well plates as described earlier. After exposure period, the HSIOs were washed with PBS and RNA was isolated using RNeasy Mini Kit (4104, Qiagen) and RT-qPCR was carried out with Power SYBR Green RNA-to-CT 1-Step Kit (4389986, ThermoFisher Scientific). The expression of *Sod1* (QT01671551, Qiagen), *Tnfa* (QT00029162, Qiagen), *Il1b* (QT00021385, Qiagen), *Capase1* (QT00001568, Qiagen) and *Axin2* (QT00037639, Qiagen) were normalized to reference gene *Gapdh* (QT00079247, Qiagen). Relative expression was determined using the ΔΔCt method. The statistical analysis was performed on ΔCt values.

### Caspase 3/7 and Caspase 1 Activity Measurements

HSIOs and HIEC-6 cells were treated as described earlier. The Caspase-Glo 3/7 Assay System (G8090, Promega) was used to measure Caspase 3 and 7 activity for both organoids and monocultures. The Caspase Glo 1 Inflammasome assay (G9951, Promega) kits were employed to measure Caspase 1 levels for HIEC-6 cells as per the manufacturer’s instructions.

### FerroOrange Staining

HIEC-6 cells were plated at a density of 31,250 cells/well in 24-well plates and treated with 1 × 10^11^ particle/mL of NPPP-1. After 96 h of exposure, the cells were washed twice with Hanks’ Balanced Salt Solution (HBSS, 14025092, ThermoFisher Scientific) and incubated for 30 min with 1 uM BioTracker FerroOrange Live Cell Dye (SCT210–35NMOL, Sigma-Aldrich) in HBSS. Then the cells were washed twice with HBSS and imaged on a EchoRevolve R4 (Discover Echo) fluorescent microscope. The mean intensity per cell was calculated using CellProfiler 4.2.8 (Broad Institute).

### μ- Infrared Spectroscopy (μ-IR)

Infrared hyperspectral imaging was performed using a Bruker Hyperion II—quantum cascade laser (QCL) based infrared laser imaging microscopy (ILIM) with a 15×/0.4 NA objective in reflection geometry. The QCL source consisted of 4 tunable external cavity lasers covering a frequency range of 950–1900 cm^–1^ coupled with a room-temperature microbolometer detector with a 200 × 200-pixel focal plane array. Hyperspectral images were recorded at a resolution of 4 cm^–1^ with a coaddition of 3 scans.

10 μm frozen sections of HSIOs and 3D skin models (as described in ref 40) were placed on IR-reflective slides (MirrIR Corner Frosted, 1 × 3 in., Kevley Technologies) and incubated at 37 °C for 5 min to allow for adhesion. The slides were then incubated in PBS at room temperature for 10 min to remove OCT. Finally, the slides were washed with Milli-Q water for 10 min and air-dried overnight. For HIEC-6, the cells were trypsinized (07901, StemCell Technologies) to obtain a single cell suspension and then seeded onto IR-reflective slides using a Cytospin 4 (FisherScientific) operated at 1000 rpm for 5 min.

Spectra in the ILIM hyperspectral image were preprocessed with baseline correction using iterative reweighted quantile regression^41^ implemented via pybaselines. The detection of PP was accomplished using a form of anomaly detection using an autoencoder model. This was implemented in Python (v 3.12.3), using Pytorch (v2.7.0). The overall approach was to train an autoencoder model on a set of control hyperspectral images: HSIO or HIEC samples which have not been exposed to NPPP-1. The autoencoder is then used to reconstruct input spectra from images of exposed cultures. Areas of the spectrum containing sufficient signal from NPPP-1s reconstruct poorly, and these areas of are most strongly retained when the residual between the original and reconstructed spectrum are taken.

The autoencoder model consisted of an encoder consisting of 128 and 64 layers with leakyReLU activation, followed by an 8-layer bottleneck with Softplus activation, then a symmetric decoder. Residual spectra were by taking the first derivative of the difference between the original and reconstructed spectrum through a Savitzky-Golay filter, then restoring the spectrum by taking the cumulative sum in order to reduce the contribution of noise and reconstruction error. Integration of the residual spectrum between 1356 and 1390 cm^–1^ was used to generate a heatmap (μ-IR chemical mapping) of NPPP-1 content based on the 1376 cm^–1^ peak of PP, or between 1440 and 1460 cm^–1^ for a heatmap of PS content based on the 1450 cm^–1^ peak of PS.

IR spectra for the plastic materials were measured using micro-Fourier-transform infrared spectroscopy (μ-FTIR) using a Bruker Hyperion II FTIR microscope with a liquid-nitrogen–cooled MCT detector in reflection mode. Details can be found in the Supporting Information. Nine spots positioned at equal distances from each other were measured to obtain representative spectra. The carbonyl index (CI) was measured by taking the ratio in peak areas between the carbonyl peak at ∼1720 cm^–1^, and a reference peak, (CH_2_ stretching at 2720 cm^–1^).

### Atomic Force Microscopy (AFM)

For confirmation that NPPP-1 particles were present in the same tissue areas analyzed by μ-IR, the exact μ-IR slides were imaged using AFM. AFM topography images were recorded using NanoWizard II BioAFM (JPK Instruments, Berlin, Germany) mounted on an Olympus IX81 inverted microscope. AFM was performed in contact mode, and images were captured using DNP-S (Veeco/Bruker, CA) cantilever/tips with nominal spring constants of 0.06 N/m and a set point of 1 V. Following preliminary scans of larger areas (50 μm × 50 μm–100 μm × 100 μm) f-view and guided by μ-IR heatmaps, multiple smaller areas (5–20 μm) were recorded with a 512 pixel × 512-pixel resolution and a scan rate of ∼1 Hz. AFM images were processed and analyzed using JPK DP Processing (version 5.1.8).

AFM topography images of SynPP were recorded using the same instrument with HQ: XSC-11/Al BS tips (Cantilever B, MikroMasch, CA, USA) with a nominal spring constant of ∼2.7 N/m and a resonance frequency of ∼80 kHz under intermittent contact mode. Samples for AFM were prepared by drop-casting 20 μL of SynPP dispersion onto freshly cleaved 1 in. by 1 in. mica substrates. Following preliminary scans of larger areas (50 μm × 50 μm–100 μm × 100 μm) for sample overview, multiple smaller areas (5–20 μm) were recorded with a 512 pixel × 512-pixel resolution and a scan rate of ∼1 Hz.

### Statistical Analysis for Biological Characterization

Unless otherwise stated, all the experiments described here are from three independent biological replicates, with three technical replicates per condition within each biological replicate. Biological replicates represent independent cell or organoid preparations, and technical replicates represent repeated measurements within a given preparation. Data were analyzed using GraphPad Prism 8 software. Unpaired *t* tests were done for single comparisons between two groups. A one-way analysis of variance (ANOVA) was conducted for more than two groups, followed by a posthoc test and adjustment for multiple comparisons. A value of *p* < 0.05 was set as the limit of statistical significance.

## Results and Discussions

### Material Specific Viability Responses of Intestinal Models to NPPP-1, Synthetic PP and Aged Synthetic PP under Matched Particle-Number Exposure

Exposure of organs and tissues to NPs that have been weathered via environmental conditions is emerging as a toxicological concern. We applied laser ablation to bulk PP polymers to produce an environmentally relevant nanoscale PP reference material NPPP-1 that mimicked the heterogeneous size distribution and displayed an irregular disc/flake like morphology characterized via a high aspect ratio (diameter/height).^13^ In addition, this reference material also exhibited thermo-oxidative traits similar to environmentally weathered plastics (Figure 1A). FTIR spectra revealed clear chemical differences between bulk PP used as the source material (PP source) for laser ablation (red), environmental PP fragments (PP-Env) (blue, collected from waste samples, which were possibly exposed in ambient conditions for decades), and the laser-machined NPPP-1 (green) (Figure 1A). Bulk PP exhibited the characteristic isotactic PP bands at 2920–2840 cm^–1^ (CH_3_/CH_2_ stretching), 1455, 1375 cm^–1^ and 1167–997 cm^–1^ (CH_2_/CH_3_ bending), with no detectable carbonyl absorption. In contrast, both NPPP-1 and PP-Env exhibited a distinct carbonyl peak at ∼1720 cm^–1^, consistent with photo-oxidative oxidation of PP (C=O stretching) and indicative of the presence of carbonyl-containing functional groups (Figure 1A). The emergence of this band in NPPP-1 demonstrates that the laser-machining process induces surface oxidation similar to that observed in naturally weathered PP. Although, IR analysis of the PP bulk materials did not reveal signatures of major additives or fillers, these techniques are not sensitive to trace amounts of all known or proprietary additives. Thus, supplier dependent differences in residual additives between bulk PP used for NPPP-1 and SynPP cannot be fully excluded and may contribute to material specific biological effects.

**Figure 1:**
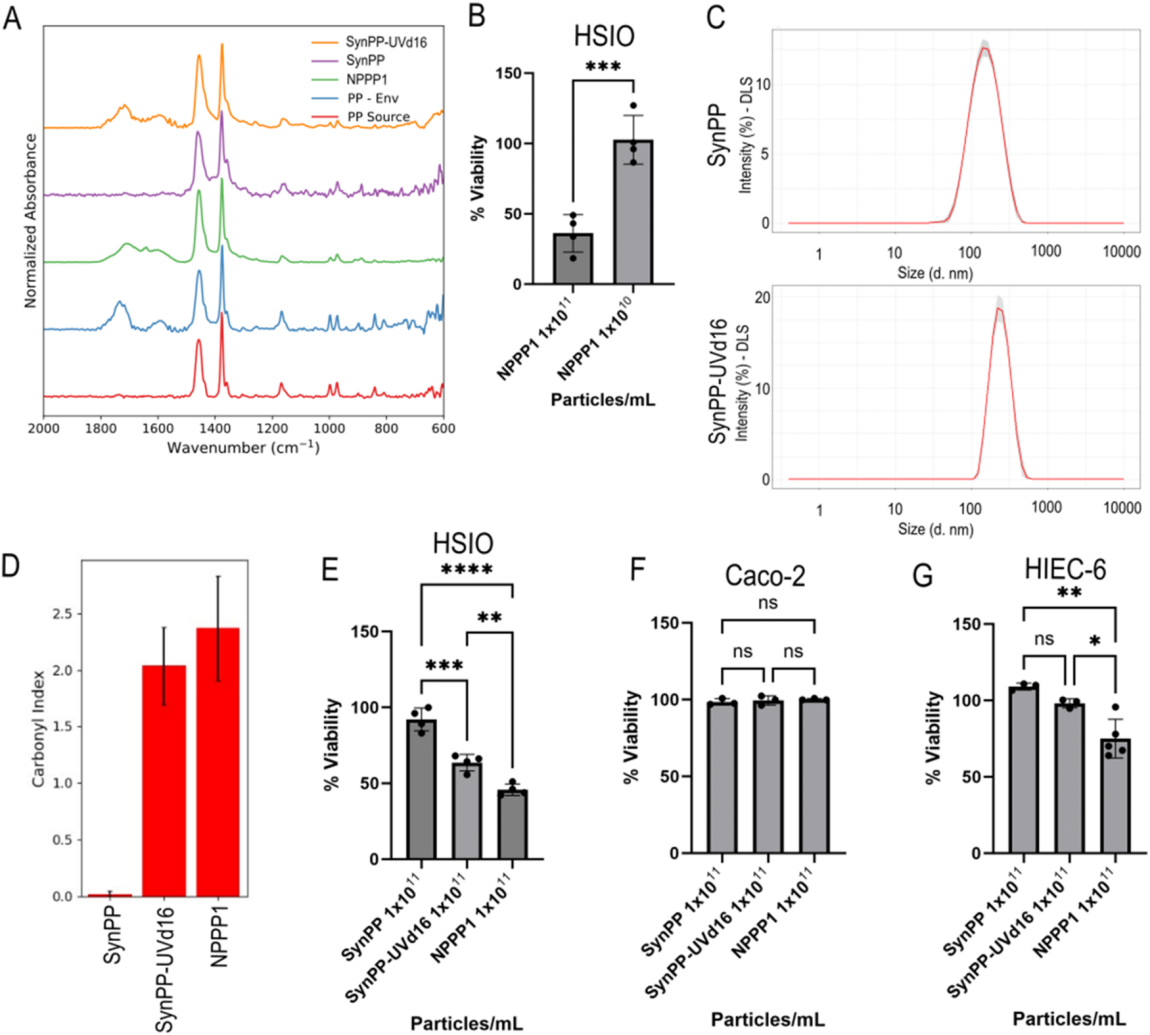
Material-specific effects of NPPP-1 and SynPP variants on the viability ofintestinal epithelial models. (A) FTIR spectra of original source PP (PP Source) (red), environmental PP fragments (PP-Env) (blue), SynPP (purple), SynPP-UVd16 (orange) and NPPP-1 (green). (B) Relative viability of HSIOs treated with NPPP-1 at concentrations of 1 × 10^11^ and 1 × 10^10^ particles/mL. (C) Particle size distribution of SynPP (top) and SynPP-UVd16 (bottom) measured via DLS. (D) Measurement of carbonyl index of SynPP and SynPP-UVd16. (E–G) Relative viability of HSIOs (E), Caco-2 (F) and HIEC-6 cells (G) treated with 1 × 10^11^ particles/mL of NPPP-1, SynPP and SynPP-UVd16. Values on each graph are shown as mean ± SD of three independent experiments each with replicates (*n* = 3). Statistical significance was determined with one-way ANOVA. *P* < 0.05 was considered significant. *P < 0.05; **P < 0.01; ***P < 0.001; ****P < 0.0001. *P* > 0.05, ns: not significant.

The exposure concentrations for NPPP-1 were selected using a biological effect guided principle, with particle number concentrations quantified using NTA. Preliminary screening indicated that 1 × 10^10^ particles/mL did not significantly affect cell or organoid viability under the tested conditions, whereas 1 × 10^11^ particles/mL produced consistent responses suitable for mechanistic analysis. These concentrations were not intended to directly represent environmental exposure levels but rather to enable detection of biologically meaningful effects. Consistent with this design, exposure of HSIOs to NPPP-1 elicited a notable reduction of viability in a dose-dependent manner (Figure 1B) similar to our observations using murine intestinal organoids,^13^ where NPPP-1 treatment showed a trend toward reduced viability (∼55%) at the highest concentration tested. Compared to NPPP-1 at a concentration of 1 × 10^10^ particles/ml, the HSIOs treated with 1 × 10^11^ particles/mL (∼9.6 μg/mL; mass-equivalent calculation based on certified total carbon and measured particle number is provided in the Supporting Information) showed approximately ∼3-fold decrease in viability as measured via quantification of ATP levels (Figure 1B).

To compare the effect of environmentally mimetic laser ablated NPPP-1 to that of NPs obtained via synthetic chemistry, we generated two different PP nanoparticles: (1) SynPP, a pristine spherical PP nanoparticle generated synthetically via organic solvent precipitation;^39^ and (2) SynPP-UVd16, SynPP that was exposed to UVC irradiation for 16 days to facilitate accelerated aging. While UVC exposure does not replicate natural solar spectra, UVC irradiation has been widely used in laboratory settings to experimentally induce accelerated photo-oxidative surface modification of polymeric materials and NPs.^42,43^ Using DLS to characterize the size, SynPP exhibited the smallest Z-average hydrodynamic diameter (136 nm) with a moderate polydispersity index (PDI = 0.16), consistent with a relatively narrow and uniform particle population. SynPP-UVd16 showed a substantially larger hydrodynamic size (229 nm) and a markedly lower PDI (0.08), indicating the appearance of a more monodisperse subpopulation after UV exposure (Figure 1C and Table 1). In contrast, the environmentally relevant NPPP-1 showed a larger size (283 nm) accompanied by a significantly higher degree of heterogeneity (0.25), reflecting the broader size distribution expected from real-world mixed-source plastic particulates (Table 1).^12^ All the plastic materials exhibited negative zeta potentials under the measurement conditions, indicating colloidal stability and surface-charge differences (Table 1). Beyond changes in particle size, exposure to UV has been shown to introduce carbonyl groups and increase the hydrophilicity of polymers.^44^ Quantification of carbonyl index, a measurand of the degree of weathering, indicated SynPP-UVd16 had more carbonyl groups present compared to SynPP (Figure 1A,D). At a matched particle number exposure (1 × 10^11^ particles/mL), HSIO viability responses differed across materials. Viability was approximately 92% for SynPP (∼173 μg/mL; Supporting Information), 63% for SynPP-UVd16 (∼37.6 μg/mL; Supporting Information), and 45% for NPPP-1 (∼9.6 μg/mL; Table S1) (Figure 1E). These differences should be interpreted as material-specific responses rather than a direct toxicity ranking, given the substantial variation in mass concentrations and surface chemistry. This aligns with previous work that has shown weathered or UV-degraded plastic fragments exhibit stronger interactions with biological membranes and elevated ROS generation compared to pristine particles.^45–47^ We have now extended those insights into a more biologically relevant human intestinal microenvironment model, where laser ablation generated nanoscale plastics with oxygen-containing groups (Figure 1A,D) likely facilitate cellular uptake and downstream toxicity. This is consistent with findings that laser ablation of PET to generate environmental NP mimics also results in cellular internalization of the particles .^47^

**Table 1:** Physicochemical Properties of PP Variants (mean ± SD, *n* = 5)

Material	*Z*-average diameter (nm)	Polydispersity index (PdI)	Zeta potential (mV)	Carbonyl index
SynPP	136 ± 3	0.16 ± 0.01	–44.9 ± 1.2	0.01 ± 0.02
SynPP-UVd16	229 ± 2	0.08 ± 0.03	–30.48 ± 0.6	2.11 ± 0.33
NPPP-1	283 ± 31	0.25 ± 0.05	–66 ± 6.4	2.58 ± 0.43

In order to validate whether these NPPP-1 mediated effects were restricted to 3D organoid models or extended to simpler 2D monolayer epithelial systems, we also carried out parallel exposures using the Caco-2, a human colorectal adenocarcinoma cell line and HIEC-6, a nontransformed primary-like intestinal epithelial cell line. These cell lines are widely used as intestinal epithelial models to study intestinal barrier function, xenobiotic toxicity, and nanoparticle uptake in 2D contexts.^48–52^ Both cell lines were treated with 1 × 10^11^ particles/mL of the PP variants. Exposure to NPPP-1, SynPP, and SynPP-UVd16 did not impact Caco-2 viability at all (Figure 1F). This is consistent with previous reports that show Caco-2 cells have a high tolerance to NPs. Cortés et al. 2020 demonstrated that PS NPs up to 100 μg/mL produced no cytotoxicity in Caco-2 cells despite measurable internalization.^53^ PP NPs derived from grinding food containers likewise elicited no cytotoxic effects in Caco-2.^54^ This relative insensitivity to polymeric NPs is likely due to their transformed origin and robust antioxidant defenses.^55^ Compared to Caco-2, the nontransformed primary like HIEC-6 cells responded to PP exposure (Figure 1G). In contrast to the HSIOs, HIEC-6 cells showed no loss of viability upon exposure to SynPP or SynPP-UVd16 (Figure 1G). However, NPPP-1 induced a clear reduction in viability (Figure 1G). It is hard to compare this finding to previous NP toxicological studies using 2D monocultures given the breadth of responses measured. For example, Sarma et al. 2022 examined the impact of PS beads and reported that epithelial cell lines only show measurable cytotoxicity and oxidative damage at relatively high (≥100 μg·mL^–1^) concentrations.^56^ This is consistent with studies showing PS sphere particles impair skin epithelial barrier integrity and trigger apoptosis in monolayer cultures at doses exceeding typical environmental relevance.^36^ However, other studies have shown that translocation of NPs across epithelial layers using advanced 2D lung and intestinal barrier models did not exhibit pronounced loss of viability.^57^ Overall, these findings suggest that HSIOs, with their biomimicking multicellular composition and structural complexity, may be more sensitive to subtle surface modifications, whereas 2D epithelial models like HIEC-6 only mount strong responses to the more dramatically altered surface chemistries produced by laser ablation for NPPP-1.

In addition to environmental surface chemistry, NPPP-1 also exhibits irregular disc/flake-like shape.^13^ In contrast, SynPP and SynPP-UV-d16 particles retain a predominantly spherical particle shape, as shown by high-resolution imaging (Figure S1), indicating minimal shape deformation following exposure to UV aging conditions. Representative images reveal discrete and rounded particles with preserved curvatures in both SynPP and SynPP-UVd16 samples, despite limited particle aggregation (Figure S1). The combined effect of morphology and surface oxidation likely enhances NPPP-1 internalization under the exposure conditions tested, consistent with differences in particle shape and surface chemistry rather than intrinsic toxicity. Nanoparticle shapes such as spherical vs flat disc-like morphologies, can influence cellular uptake and endocytosis.^58,59^ Several studies have reported that nonspherical or angular particulates display greater cell surface adhesion and internalization compared to spheres. For example, particles with higher aspect ratio or sharp angular features adhere more and are more readily internalized.^60^ Similar observations have been made with gold nanoparticles, with triangular/anisotropic gold particles outpacing spheres for uptake in murine macrophages.^61^ Irregular and fragmented plastics tend to produce a greater oxidative and inflammatory response compared to spherical particles.^62^ Smaller and fragmented plastics elicit a greater toxicity and mortality in marine zooplanktons than spherical plastic particles.^63^ Given these past results, it is expected that particle shape can impact cellular processes and viability. In addition to surface oxidation and irregular morphology, concentration of particles was the most impactful factor governing biological outcomes as HSIOs showed a consistent dose dependent decrease in viability.

### NPPP-1 Induces Redox Imbalance, DNA Damage, and Inflammatory Signaling

Generation of ROS is a well-recognized mechanism underlying NP-mediated cytotoxicity across diverse cell types and organisms. Excessive levels of ROS can trigger a cascade of downstream responses, including DNA damage, inflammation, apoptosis, lipid peroxidation and ferroptosis.^21,64–66^ MPs have been shown to activate mitochondrial ROS which led to oxidative damage and lipid peroxidation of spermatogonia, ultimately reducing fertility.^67^ Similar observations in *Daphnia pulex* link NP exposure to ROS-driven inflammation, developmental impairments, and reproductive toxicity.^68^ Given the distinct and more consistent phenotype and the study’s focus on an environmentally mimetic PP reference material, downstream mechanistic analyses were prioritized for NPPP-1; and SynPP and SynPP-UVd16 were excluded from further analysis. Consistent with prior reports linking plastics to ROS-driven oxidative damage and inflammation, we found that NPPP-1 induced a pronounced elevation of ROS generation in HSIOs. Compared to untreated controls, ROS levels in cells exposed to 1 × 10^11^ and 1 × 10^10^ particles/mL were elevated by approximately 45-fold and 20-fold, respectively (Figure 2A).

**Figure 2:**
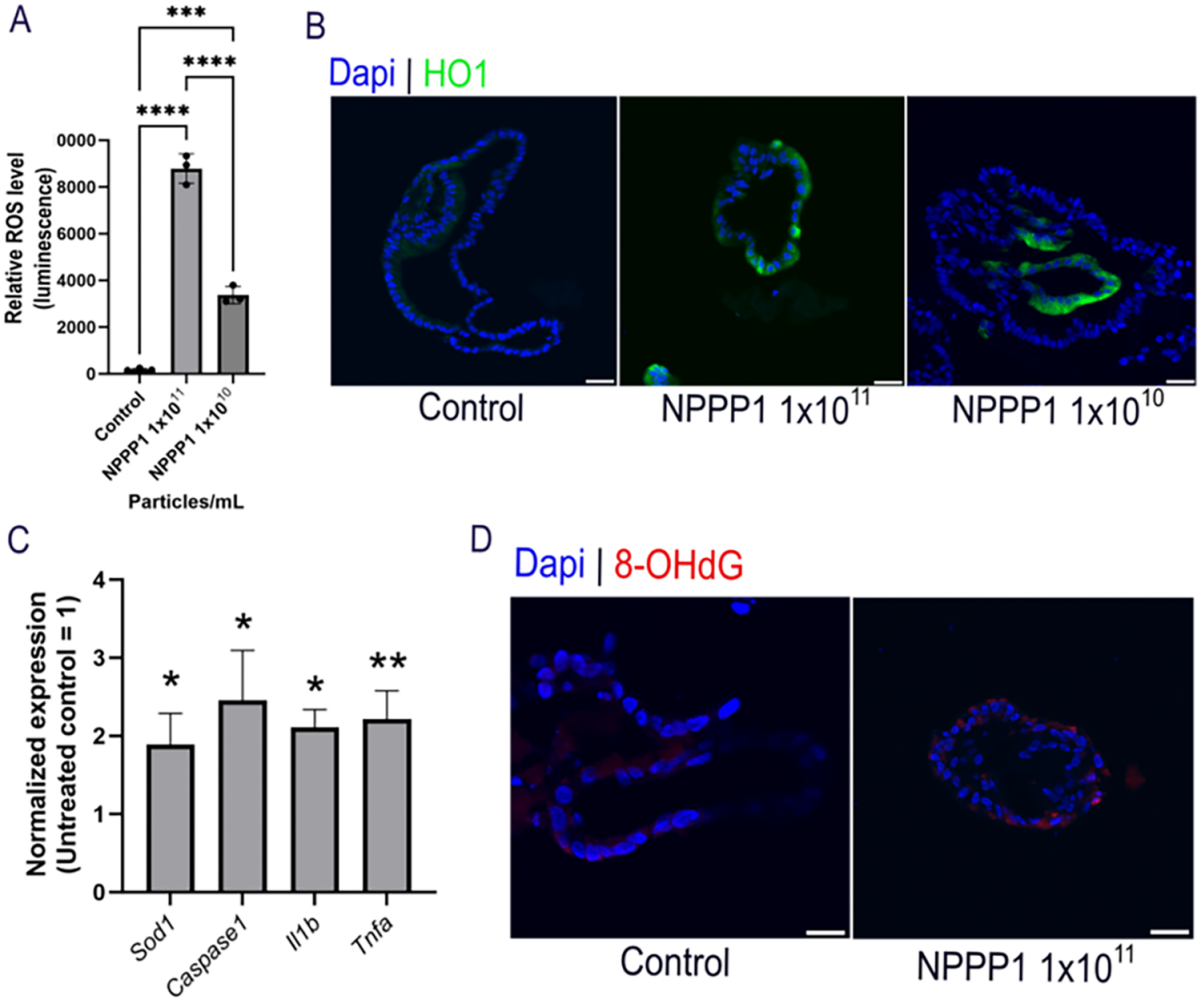
NPPP-1 reference material induces oxidative stress, inflammation and DNA damage. (A) Relative ROS levels as measured via ROS-Glo assay in HSIOs treated with NPPP-1. (B) Immunofluorescence of HO1 (green) in HSIOs treated with NPPP-1. Nuclei is stained in blue (Dapi). Scale bars measure 30 μm. Images are representative of three biological replicates (*n* = 3), with 5 HSIOs analyzed per replicate. (C) Relative mRNA levels for *Sod1*, *Caspase1*, *Il1b* and *Tnfa* in HSIOs treated with NPPP-1. Values on each graph are shown as mean ± SD of three independent experiments each with replicates (*n* = 3). Statistical significance was determined with one-way ANOVA. *P* < 0.05 was considered significant. *P < 0.05; **P < 0.01; ***P < 0.001; ****P < 0.0001. *P* > 0.05, ns: not significant. (D) Immunofluorescence staining showing cells with mitochondrial DNA damage (8-OHdG in red) in NPPP-1 treated HSIOs. Nuclei is stained in blue (Dapi). Scale bars measure 30 μm. Images represent three biological replicates (*n* = 3), with 5 HSIOs analyzed per replicate.

At the protein level, heme oxygenase1 (HO1), a cytoprotective stress-inducible enzyme commonly used as a marker of oxidative stress,^69,70^ was strongly induced in HSIOs in a concentration dependent fashion. Control HSIOs exhibited negligible HO1 signal, whereas treatment with 1 × 10^11^ particles/mL produced more intense signal along the entire epithelial layer (Figure 2B). In the 1 × 10^10^ particles/mL condition, HO-1 signal was detectable in discrete regions of the epithelial layer, indicating partial or localized induction of oxidative-stress response. (Figure 2B). Given that the most significant response was observed with a high concentration of 1 × 10^11^ particles/mL, subsequent analysis was performed only for HSIOs treated with this concentration. As exposure to NPs have been shown to upregulate antioxidant enzymes such as superoxide dismutase 1 (Sod1) and activate inflammatory-Caspase1/interleukin1 β (Il1β)/ tumor necrosis factor α (Tnfα) axes in epithelial cells,^1,5,71^ we selected genes such as *Sod1*, *Caspase1*, *Il1b* and *Tnfa* as molecular markers of oxidative-inflammatory stress in our study. Gene expression profiling revealed robust transcriptional activation of these genes (Figure 2C). Elevated *Sod1*, an antioxidant defense response,^72^ validated the ROS functional assay (Figure 2A) and HO1 immunofluorescence (Figure 2B). In addition, mRNA expression of *Tnfa* and *Il1b* for inflammatory cytokines was upregulated as well, indicating NPPP-1 mediated pro-inflammatory environment^73,74^ (Figure 2C). Induction of *Caspase1* suggested inflammasome activation^75^ (Figure 2C).

We also examined the downstream effect of this oxidative stress by performing immunofluorescence for 8-OHdG, a predominant form of free-radical-induced oxidative lesions that is employed as a marker of oxidative DNA damage.^76^ Environmentally persistent free radicals on plastics generated via photoaging processes have been shown to increase levels of 8-OHdG.^77^ Immunostaining revealed that 8-OHdG appeared along the epithelial perimeter of NPPP-1 treated HSIOs, localized predominantly in the perinuclear regions consistent with mitochondrial oxidative DNA damage whereas the control HSIOs showed almost no signal (Figure 2D). These findings are consistent with the broader literature, which recognizes ROS increases caused by NPs as key mediators of cellular toxicity capable of damaging lipids, proteins, and nucleic acids, ultimately leading to mitochondrial dysfunction and activation of stress-signaling cascades.^78,79^ For instance, Woo et al. demonstrated that exposure to PP NPs can activate p38-MAPK-dependent NF-κB pathway and induce ROS-mediated mitochondrial damage, linking oxidative signaling to inflammation.^1^ Similarly, Hou et al. implicated NPs in the induction of oxidative stress and inflammatory injury in colon and intestinal organoids, supporting a common mechanism in which oxidative stress likely precedes both cellular degeneration and cytokine activation.^80^

### NPPP-1 Triggers Regulated Cell Death Pathways in HSIOs

Building on the evidence that NPPP-1 induces overwhelming ROS accumulation and oxidative DNA damage, we next investigated which RCD pathways contributed to the observed loss of viability, focusing on those known to be sensitive to nanoparticle exposure. RCD encompasses diverse mechanisms, including apoptosis, ferroptosis, necroptosis, pyroptosis, autophagy-dependent cell death, parthanatos, entosis, lysosome-dependent cell death.^81^

To assess whether ferroptosis played a role in NPPP-1-induced cytotoxicity, we quantified malondialdehyde (MDA) levels, a reactive aldehyde generated through ROS-driven peroxidation of polyunsaturated fatty acids (PUFAs) in cell membranes. Elevated MDA is a reliable indicator of lipid peroxidation and ferroptotic injury. MDA was evaluated at the high concentration of 1 × 10^11^ particles/mL of NPPP-1 only, as lower concentrations (1 × 10^10^ particles/mL) did not significantly affect HSIO viability under the tested conditions (Figure 1B). In our analysis, MDA concentrations were measured using a competitive ELISA assay. NPPP-1 treatment resulted in a 2.5-fold increase in MDA compared with untreated controls (Figure 3A), consistent with NPPP-1–mediated overproduction of ROS (Figure 2A), which drives PUFA peroxidation.^23^

**Figure 3:**
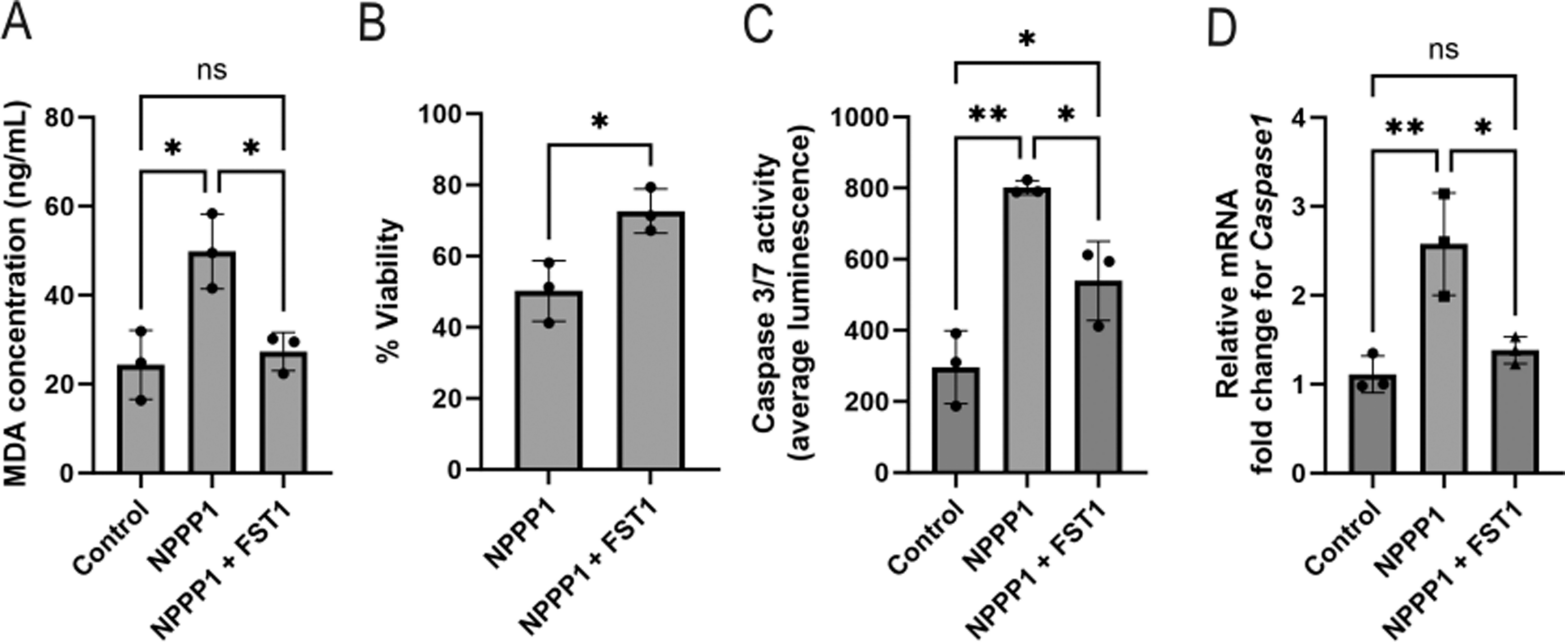
Treatment with NPPP-1 induces ferroptosis, apoptosis and pyroptosis in intestinal models. (A) Concentration of MDA in the lysates of HSIOs treated with NPPP-1 and NPPP-1 with FST1. (B) Relative cell viability of HSIOs treated with NPPP-1 and NPPP-1 with FST1. (C) Apoptosis measured via caspase 3/7 activity in HSIOs treated with NPPP-1 and NPPP-1 with FST1. (D) Relative mRNA levels of *Caspase1* of HSIOs after treatment with NPPP-1 and NPPP-1 with FST1. Values on each graph are shown as mean ± SD of three independent experiments each with replicates (*n* = 3). Statistical significance was determined with *t*-test (two groups) and one-way ANOVA (three or more groups). *P* < 0.05 was considered significant. *P < 0.05; **P < 0.01; ***P < 0.001; ****P < 0.0001. *P* > 0.05, ns: not significant.

Upregulation of antioxidant defenses (HO1, *Sod1*) observed earlier (Figure 2B,C) underscores that cells in HSIOs attempt to counteract this lipid peroxidation; however, excessive ROS (Figure 2A) likely overwhelm this protective capacity, tipping the balance toward cell death. These findings are consistent with reports that NPs can trigger ferroptosis, characterized by intracellular labile Fe^2+^ accumulation and lipid peroxidation.^25,64^ Wu et al. observed increased MDA in human lung bronchial epithelial cells treated with 100 nm PS particles at 100–400 μg/mL,^64^ while porcine oocytes exposed to 100 nm PS also showed elevated intracellular Fe^2+^ and MDA levels.^65^

To further confirm that ferroptosis was a significant contributor to cell death, we treated the cells with the ferroptosis inhibitor Ferrostatin-1 (FST-1)^82^ which partially recovered cell viability, from approximately 50% in NPPP-1- treated HSIOs to 72% in the NPPP-1 + FST1 treated HSIOs (Figure 3B). This partial rescue indicates that ferroptosis is a major contributor to NPPP-1–mediated cytotoxicity but that additional RCD pathways, such as apoptosis or pyroptosis, may also be involved.

Consistent with results in HSIOs, we found in HIEC-6 epithelial cells, that NPPP-1 exposure resulted in an increased concentration of MDA (Figure S2A). This was also accompanied by an increase in the intensity of FerroOrange, a fluorescent dye that binds selectively to labile intracellular Fe^2+^, indicating increased levels of intracellular labile Fe^2+^, which is another key hallmark of ferroptosis (Figure S2B–C).^24^

To better determine what other RCD pathways, contribute to the reduction in cell viability, Caspase 3/7 activity was measured using a luminescence assay to evaluate apoptosis and Caspase1 mRNA levels were measured to evaluate pyroptosis. Caspase 3 and 7 are effector cysteine-aspartate proteases activated by initiator caspases (8 or 9) that mediate apoptotic dismantling.^83^ NPPP-1 exposure increased Caspase 3/7 activity by more than 2-fold, which decreased but did not return to basal levels following FST-1 cotreatment (Figure 3C). This persistence suggests that apoptosis remains active even when ferroptosis is partially inhibited. Similarly, *Caspase1* mRNA levels (a pyroptosis biomarker) increased with NPPP-1 exposure and were reduced by FST-1 treatment, returning to near untreated control levels (Figure 3D). In addition to apoptosis, pyroptosis has emerged as an NP-mediated inflammatory RCD initiated by inflammasome activation. Upon activation, inflammasome adapters such as NLRP3 recruit procaspase-1, which cleaves pro-IL-1β into mature cytokines that drive gasdermin D–dependent cell lysis and inflammation.^84^ Prolonged PS exposure in murine models has been linked to pyroptotic hepatic damage via NLRP3 activation and caspase-1–dependent IL-1β release.^85^ In vitro studies with THP-1 monocytes also show robust NLRP3/caspase-1 activation following nanoparticle exposure.^86^

Emerging evidence indicates that ferroptosis can interact with both apoptosis and pyroptosis under certain pathological conditions. Excessive lipid ROS generated during ferroptosis can activate inflammasomes and induce pyroptosis-like features especially in epithelial and immune cells.^87,88^ Crosstalk between ferroptosis and apoptosis has also been documented; overexpression of the antiapoptotic protein Bcl-2 can alleviate ferroptosis through mitochondrial ROS downregulation,^89^ and early apoptotic markers appear in cells treated with the ferroptosis inducer erastin.^90^ Together, these findings suggest that the loss of viability in NPPP-1 exposed HSIOs arises from combined activation of ferroptosis, apoptosis, and pyroptosis.

Like HSIOs, HIEC-6 cells also exhibited heightened (∼2-fold) Caspase 3/7 activity (Figure S2D); however, no Caspase 1 activation was detected (Figure S2E), highlighting potential context-dependent activation of pyroptosis. These results are consistent with recent studies showing coactivation or sequential engagement of multiple RCD pathways.^91^

Multiple studies corroborate that nanoparticles can initiate various RCD pathways-including ferroptosis, apoptosis, and pyroptosis-across biological systems. PS particle exposure induces apoptosis in human cervical cancer and murine granulosa cells through DNA damage,^92,93^ while also promoting lipid peroxidation and Fe^2+^ dysregulation (hallmarks of ferroptosis) in lung epithelia.^64^ Comprehensive reviews suggest that ferroptosis often serves as the initiating event in particle-mediated cytotoxic cascades, followed by apoptosis once antioxidant defenses are exhausted, whereas pyroptotic signatures are more frequent in immune or multicellular contexts than in simple 2D epithelia.^91^

Taken together, our data show that NPPP-1 exposure is consistent with ferroptosis engagement as an early dominant stress response, with apoptosis emerging as a secondary program in epithelial cells. Caspase 1 activation in in HSIOs but not in HIEC-6 suggests that pyroptosis may require a more complex tissue context to manifest.

A formal dose response and time-course analysis of ferroptosis markers was not performed in this study. Mechanistic assays were prioritized for the biologically effective NPPP-1 exposure condition (1 × 10^11^ particles/mL), as lower concentrations did not significantly impact organoid viability under the tested exposure duration. Accordingly, the present data support ferroptosis involvement under these conditions but do not resolve the full temporal or concentration dependence of this response.

### Direct In Situ Evidence of Environmentally Relevant NPPP-1 Uptake in Human Organoids and Epithelial Cells

The detection of NPs in complex biological samples, such as tissues and organoids, especially with label-free plastics, remains a challenge. While fluorescently labeled plastics are commonly employed to track the plastics in biological systems, it is increasingly recognized that this approach can introduce significant artifacts. The conjugation or incorporation of dyes may modify particle size, charge, and surface chemistry, thereby altering uptake dynamics and interactions with cells and tissues.^94,95^ Several studies have also demonstrated that fluorescent signals may not reliably indicate the presence of plastic, as these dyes can leach from the plastic matrix and redistribute into lipids or tissues, producing false positives. For example, experiments performed with the nematode *Daphnia magna* showed that the fluorescence localized in lipid droplets originated from leached dye rather than uptake particles.^96^ Nile red, a commonly used dye, is prone to nonspecific staining of natural organic matter and may lead to inflated counts of MPs and NPs.^97^ Conventional transmission electron microscopy (TEM) also struggles with soft, low-electron-density polymers, such as PP. In addition, polymers are also prone to beam damage, limiting its utility in the identification of nonlabeled NPs in tissues.^98,99^

To corroborate that the observed cytotoxic and ferroptotic responses were linked to NPPP-1 uptake, we used a correlated imaging technique: μ-IR imaging by ILIM and AFM to track NPPP-1 in the HSIOs. This complementary imaging workflow was applied to 10 μm frozen sections of HSIOs exposed to NPPP-1. The ILIM technique produces hyperspectral infrared images with polymer-specific spectral fingerprints directly in cells and tissue.^100^ While ILIM has a diffraction-limited spatial resolution (∼10 μm) that cannot resolve plastics at the nanoscale, it is label free and nondestructive. The unique chemical signature of PP is identifiable once the localized concentration is high enough. This high concentration is also more readily detected with higher-resolution AFM imaging, allowing for coregistration of IR and AFM images in a region of interest (ROI) on the same sample. AFM offers topography with high spatial resolution that complements the chemical specificity of μ-IR.^99^

Before applying ILIM to detect NPPP-1 within HSIOs, we first validated our imaging and analysis workflow using a biological positive control. We used a previously established 3D skin model composed of primary keratinocytes, fibroblasts, and THP-1–derived macrophages.^40^ This model was shown to support topical deposition and penetration studies of exogenous materials, including polystyrene beads, though μ-IR imaging to assess particle uptake was not used previously. In the present study, we employed the same tissue construct and exposed it to 100 nm polystyrene beads to generate a reference sample for spectral validation. The μ-IR imaging detected characteristic polystyrene peaks (aromatic C–H and C=C bands) predominantly on the epidermal surface and in certain regions of the dermis (Figure S3), confirming that the detection pipeline is suitable for identifying polymer particles within tissue sections.

For HSIOs exposed to NPPP-1 at 1 × 10^11^ particles/mL concentration, the IR spectra revealed discrete hotspots matching spectral bands characteristic of PP, which were consistent with reference spectra of NPPP-1 (Figure 4C). To improve the visibility of these signals from the broad biological background, a machine-learning driven background-subtraction process was used to identify the PP IR peaks which are anomalous from the control samples (more details can be found in the Methods section). Through this approach, a processed spectrum: the residual between the original spectrum and a predicted background signal, is produced (Figure 4C). In areas containing NPPP-1, peaks characteristic of polypropylene C–H bending at 1376 cm^–1^ and 1456 cm^–1^ can be observed. Integrating the area of one of these peaks yields a heatmap, a visual representation of the PP presence by IR (Figures 4A and S4A) if the local NPPP-1 concentration is high enough to produce a significant absorption signal above the background. Through this machine-learning driven signal processing technique, narrow absorption signals unique to PP was able to be cleanly distinguished from the broader biological background down to the signal-to-noise level of the measurement (Figure 4A,C). The AFM imaging (Figure 4B,D-1,E-1 and S4B–D) of the corresponding area (area denoted as “B” in Figure 4A and the area denoted in S4A) shows some bright features (Z-scale of about 1- 2 μm) matching hotspots in the IR heatmap (Figures 4A and S4A). Higher resolution AFM imaging of different subregions (Figure 4E,E-1 and S4B,C) confirmed the presence of submicron particulate structures embedded within the epithelial surface, which colocalized with the μ-IR-identified regions (Figures 4A and S4A). These areas with particulate structures were regularly correlated with a strong IR signal for PP as shown in the selected IR absorbance spectrum (Figure 4E-3). Although the sample prepared by a microtome was not atomically flat enough to provide a baseline for particles’ height analysis by AFM, a cross-section profile indicates that particulates have sizes of submicrons assuming a sphere shape (Figures 4E-1, E-2 and S4C,E). These sizes of particulates are significantly larger than that of individual NPPP-1. It is well-known that nanoparticles tend to cluster when they are up-taken by cells. Nevertheless, the size of the particulates on these frozen sections of HSIOs exposed to NPPP-1 is not far from the mean hydrodynamic diameter measured by DLS. The AFM image in Figures 4D and S4D has very few particulate features and are confirmed to show no significant NPPP-1 uptake by IR. Overall, the synergy of these two techniques provides direct, label-free, in situ evidence of NPPP-1 uptake into HSIOs.

**Figure 4:**
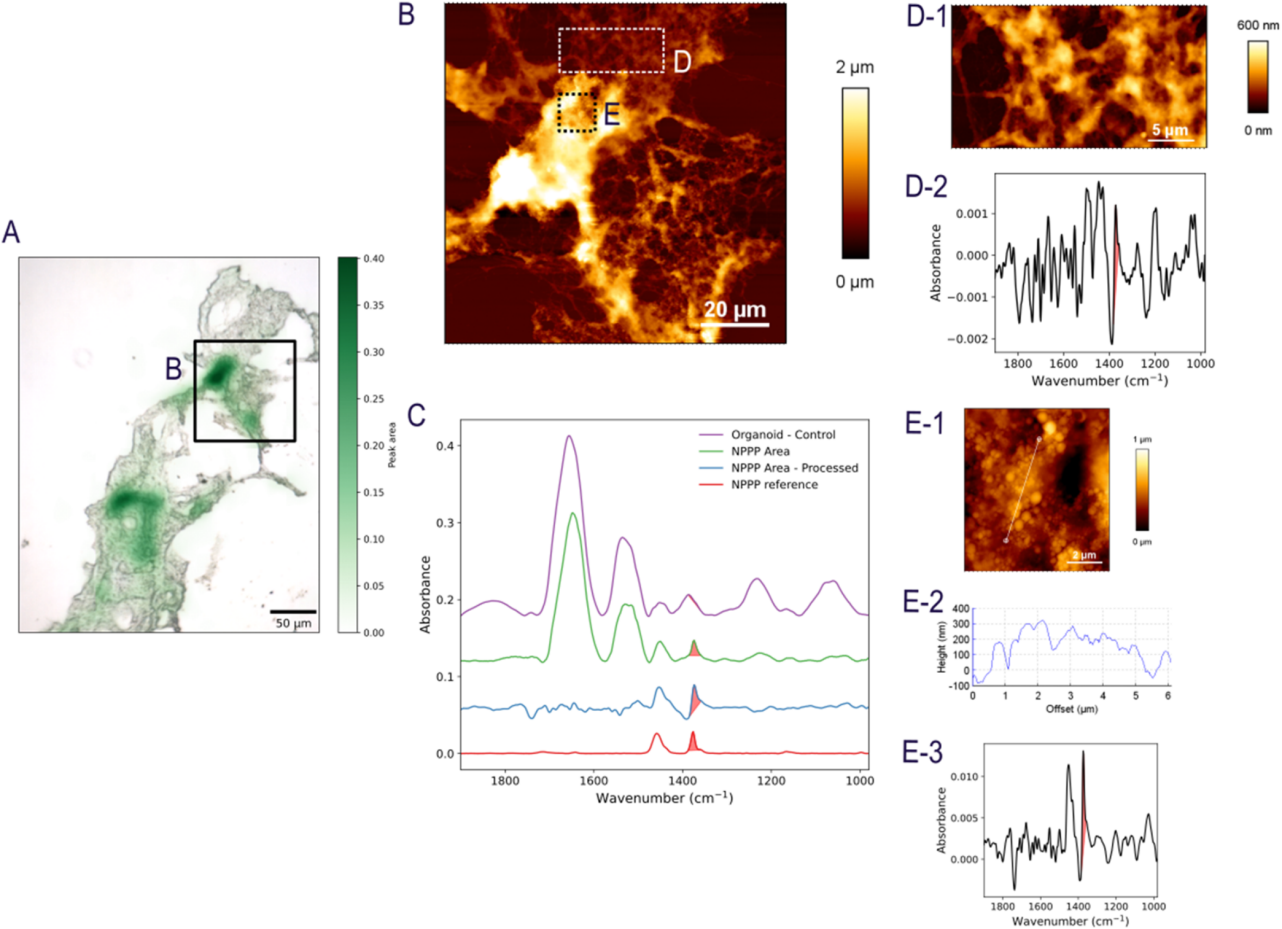
μ-IR and AFM revealed uptake of NPPP1 (1 × 10^11^ particels/mL) in the HSIOs. (A) Heatmap from μ-IR imaging highlighting the location of PP signal (hotspots in green overlaid on visual image) and uptake in HSIO 3D organoids. Image is created by removing the IR signal of the biological background using an autoencoder, and then integrating the peak at 1377 cm^–1^, as shown in the infrared spectra in (C). (B, D, E) Validation of PP uptake by AFM in the area denoted in image A. (D) Areas without PP do not show a strong signal for PP. (E) Areas with a strong signal for PP are confirmed to show significant NPPP uptake.

Parallel analysis performed on NPPP-1 treated HIEC-6 cells also supported these findings. NPPP-1 was observed in some cells of the HIEC-6 samples treated with the highest concentration (Figure S4F,G). Cells had characteristic peaks from proteins around 1650 cm^–1^ and 1540 cm^–1^ (Figure S4G). Additionally, the spectrum of the untreated cells has small peaks in the vicinity of the characteristic PP peaks used to identify NPPP-1. However, when recording spectra from treated cells, it is apparent that NPPP-1 peaks are much sharper and have a distinct tail, providing an identifier for PP in the cells (Figure S4F,G). The same machine-learning based signal processing technique removes the IR spectrum recorded from the control cells, and thus the remaining signal can be attributed to NPPP-1 with high confidence given the dual observations from HSIOs. Importantly, these physical data strengthen the causal link between NPPP-1 presence inside epithelial models and the downstream biological outcomes we observe.

### HSIOs Activate Wnt/β-Catenin Signaling as a Compensatory Response

Intestinal epithelia possess intrinsic regenerative capacity, often engaging pro-survival programs in response to tissue stress. Given that NPPP-1 exposure induces oxidative stress and ferroptotic injury, we asked whether compensatory signaling pathways are activated to mitigate cellular damage. Wnt/β-catenin is a key regulator of gut epithelial renewal and responds to redox imbalance.^29^ ROS can stabilize β-catenin by inhibition of GSK-3β, linking redox balance to Wnt activation.^32,101^ To investigate activation of the pathway, we performed immunohistochemistry for β-catenin and observed increased nuclear translocation of nonphosphorylated β-catenin in HSIOs treated with NPPP-1 as evidenced by greater instances of Dapi (nuclear stain) and β-catenin (green) colocalization (Figure 5A). In contrast, the β-catenin in the control HSIOs remained primarily cytoplasmic (Figure 5A). Nuclear translocation of β-catenin is a key upstream feature of canonical Wnt pathway activation.^102^

**Figure 5:**
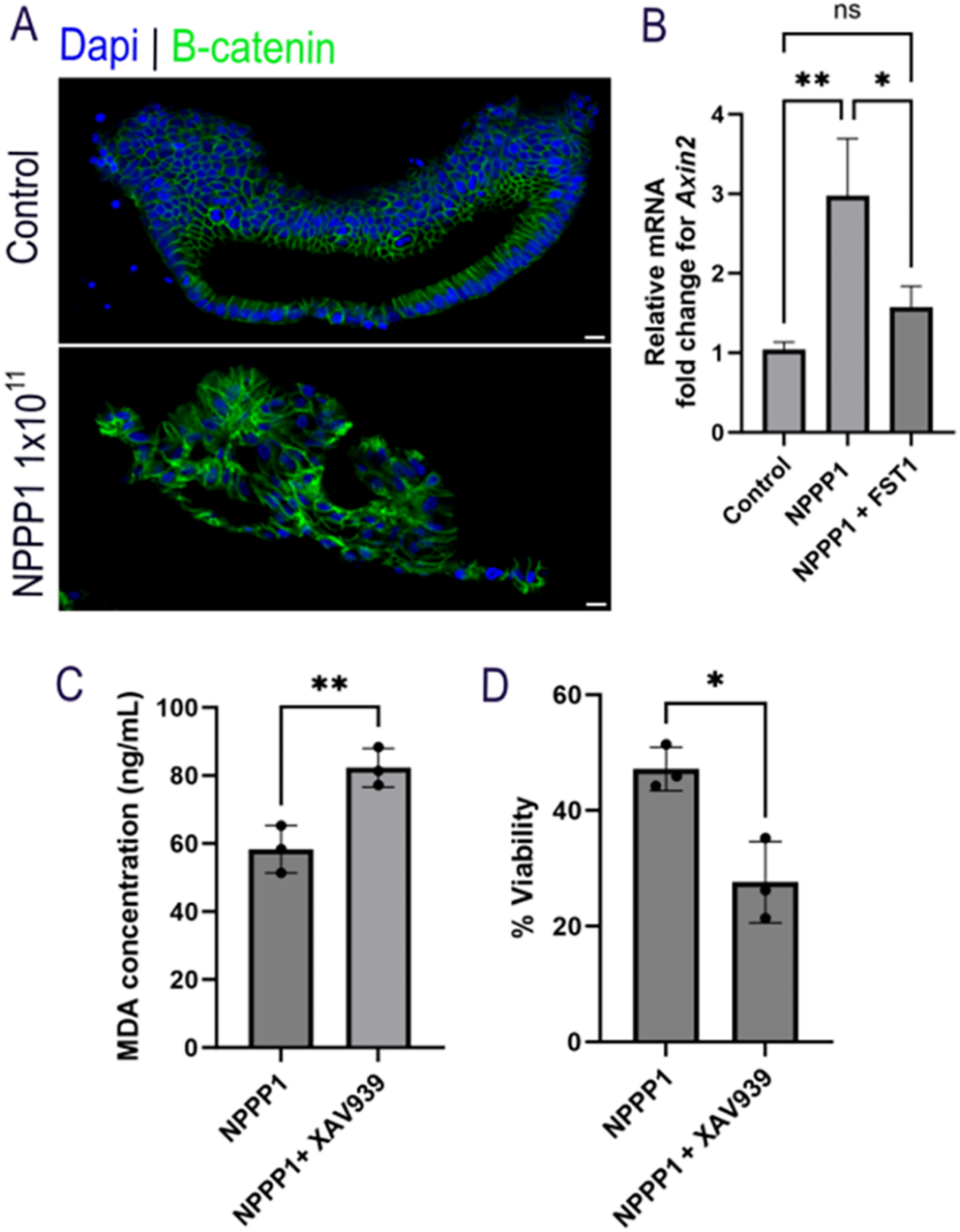
HSIOs initiate Wnt/β-catenin in response to NPPP-1 exposure to exert cytoprotective effects against ferroptosis. (A) Immunofluorescence of nonphosphorylated β-catenin (green) in HSIOs treated with NPPP-1. Nuclei is stained in blue (Dapi). Scale bars measure 20 μm. Each condition includes three biological replicates (*n* = 3), with 5 organoids analyzed per replicate. (B) Relative mRNA levels of *Axin2* in HSIOs after treatment with NPPP-1 and NPPP-1 along with FST1. (C) Concentration of MDA in HSIO lysates after treatment with NPPP-1 and NPPP-1 cotreatment with Wnt/β-catenin inhibitor XAV939. (D) Relative viability of HSIOs after treatment with NPPP-1 and NPPP-1 cotreatment with XAV939. Statistical significance was determined with *t*-test (two groups) and one-way ANOVA (three or more groups). *P* < 0.05 was considered significant. *P < 0.05; **P < 0.01; ***P < 0.001; ****P < 0.0001. *P* > 0.05, ns: not significant.

To validate transcriptional activation of the pathway, we measured the expression of downstream target gene *Axin2*. NPPP-1 exposure showed a significant 3-fold upregulation of *Axin2* gene (Figure 5B), which indicated that canonical Wnt/β-catenin signaling is activated in HSIOs post NPPP-1 exposure.^102^ Inhibition of ferroptosis by cotreatment of NPPP-1 and FST1, exhibited reduced Axin2 expression (Figure 5B), suggesting that NPPP-1 mediated Wnt activation is likely downstream of oxidative stress and lipid peroxidation^103^ and is suppressed when ferroptosis is pharmacologically blocked. A growing body of evidence also suggests that Wnt/β-catenin can buffer against ferroptosis in epithelial and cancer models by upregulating antioxidant targets. Inhibition of this pathway reduces antioxidant gene levels, leading to increased lipid peroxidation and greater sensitivity to ferroptosis.^104,105^

To further probe the functional role of Wnt/β-catenin activation in NPPP-1 mediated oxidative stress and ferroptosis, we performed a cotreatment of NPPP-1 and XAV939 (Figure 5C and D). XAV939 is a small molecule inhibitor of Wnt/ β-catenin pathway.^106^ It inhibits the enzymes Tankyrase 1 (TNKS1) and Tankyrase 2 (TNKS2), which promote degradation of the scaffolding protein Axin via poly-ADP-ribosylation. Inhibition of tankyrases by XAV939 stabilizes Axin, enhances the β-catenin destruction complex, and thereby accelerates β-catenin degradation, reducing nuclear translocation and Wnt target gene transcription.^106,107^

Pharmacological inhibition with XAV939 exacerbated lipid peroxidation as evidenced by increase of MDA levels compared to HSIOs treated with NPPP-1 alone (Figure 5C). Furthermore, this inhibition of Wnt pathway and increased lipid peroxidation further reduced viability to ∼27% (from ∼47% with NPPP-1 alone) (Figure 5D). These results indicate that Wnt/β-catenin mitigates the ferroptotic membrane damage and preserves cell viability under NPPP-1 induced oxidative stress.^106^ This is consistent with evidence of redox balance and Wnt/ β-catenin cross-talk, where oxidative cues have been shown to modulate β-catenin stability^33^ and that Wnt/β-catenin signaling confers resistance to ferroptosis by dampening lipid ROS generation.^105^

## Conclusion

Our study demonstrates that that environmentally relevant nanoscale PP, generated by laser ablation, elicit distinct biological responses in human intestinal models compared to pristine or UV-aged synthetic plastic nanoparticles. Across both 3D organoids and 2D epithelial monolayers, NPPP-1 exposure induces pronounced oxidative stress, promotes lipid peroxidation, and preferentially triggers ferroptosis, with apoptosis observed as a concurrent cell death pathway in epithelial cells. Importantly, in 3D organoids, these cellular responses are accompanied by activation of Wnt/β-catenin signaling cascade, which functions as a compensatory, pro-survival response to ferroptotic injury. By integrating biological assays with in situ imaging and spectroscopic analyses, our data provide direct evidence that environmentally relevant NPPP-1 can penetrate epithelial tissue and orchestrate a coordinated response encompassing regulated cell death and adaptive regeneration programs. Collectively, these findings highlight the critical role of particle surface chemistry—particularly oxidation associated with environmental processing—in modulating NP hazard assessment and underscore the value of human organoids as a physiologically relevant platform for dissecting molecular mechanisms and potential health risks associated with exposure to environmentally processed plastic materials.

## Supplementary Material



## Notes

μ- infrared spectroscopy (μ-IR) of nanoplastics; estimation of PP mass concentration from certified total carbon and particle number; estimated mass-equivalent concentrations for NPPP-1 exposure conditions; estimation of mass-equivalent concentrations for SynPP and SynPP-UVd16 (Table S1); both SynPP and SynPP-UVd16 particles retain a spherical shape of SynPP (Figure S1); treatment with NPPP-1 induces ferroptosis and apoptosis in intestinal epithelial cells (Figure S2); ILIM can be employed to detect the presence of polymers in 3D skin model (Figure S3); μ-IR and AFM microscopy revealed uptake of NPPP-1 in HSIOs and HIEC-6 cells (Figure S4) (PDF)

## Abbreviations

2D: Two-dimensional
3D: Three-dimensional
8-OHdG: 8-hydroxy-2′-deoxyguanosine
DLS: dynamic light scattering
FTIR: Fourier transform infrared
GSK-3β: glycogen synthase kinase-3 β
HO1: heme oxygenase1
HSIO: Human small intestinal organoids
Il1β: interleukin1 β
ILIM: infrared laser imaging
LRP5/6: low-density lipoprotein receptor-related protein 5/6
MDA: malondialdehyde
MP: microplastic
μ-IR: micro infrared
NLRP3: NOD-, LRR- and pyrin domain-containing protein 3
NP: nanoplastic
OCT: optimal cutting temperature
PDI: polydispersity index
PET: polyethylene terephthalate
PLA: polylactic acid
PP: polypropylene
PS: polystyrene
PUFAs: polyunsaturated fatty acids
QCL: quantum cascade laser
RCD: regulated cell deaths
ROI: region of interest
ROS: Reactive oxygen species
Sod1: superoxide dismutase 1
TANKS1: Tankyrase 1
TANKS2: Tankyrase 2
TEM: transmission electron microscopy
Tnfα: tumor necrosis factor α
UV: ultraviolet
