# Supplementary material for: Ferroptosis and Wnt/β-Catenin Signaling Triggered by Environmentally Relevant Nanoscale Polypropylene Plastics in Human Intestinal Models
Source: ACS Environ Au. 2026 Mar 18;6(3):471–86. doi: 10.1021/acsenvironau.5c00303 (PMC13195470; doi:10.1021/acsenvironau.5c00303)
Supplement: Supplementary file 1 [file vg5c00303_si_001.pdf]

Supplementary information

Ferroptosis and Wnt/β-Catenin Signaling Triggered by Environmentally Relevant Nanoscale Polypropylene Plastics in Human Intestinal Models

*Sadman Sakib**, *Maohui Chen, Stephanie Skerrett, Daniel Prezgot, Magda Vandenberg, Adrian F. Pegoraro, Zygmunt J. Jakubek, Zeina Maan, and Shan Zou**

[1]Nanoscale Measurement, Metrology Research Centre, National Research Council of Canada, Ottawa, ON K1A 0R6, Canada.

Supplementary methods

μ- infrared spectroscopy (μ-IR) of nanoplastics

IR spectra for NP were measured using a Bruker Hyperion II FTIR microscope with a liquid-nitrogen–cooled MCT detector in reflection mode with a 15×/0.4 NA objective. Environmental polypropylene fragments (PP-Env) were collected from coastal sites in Newfoundland and Labrador, Canada during shoreline plastic waste cleanup efforts. After collection, visible PP fragments were manually isolated and cleaned prior to analysis. PP-Env was included as a contextual environmental reference material to qualitatively compare the chemical features of environmentally exposed polypropylene with bulk and laboratory-generated nanoparticles. The environmental PP (PP- Env), and the original PP source material was measured with a 20×ATR objective with a Ge element. Samples were prepared on IR-reflective slides (MirrIR Corner Frosted, 1 × 3 inch, Kevley Technologies) Spectra were collected from 600–4000 cm^{-1} at 4 cm^{-1} resolution with 64 co-added scans.

Estimation of NPPP-1 mass concentration from certified total carbon and particle number

Toxicity experiments in this study were carried out using particle number concentration (particles/ mL). To facilitate comparison with prior nanoplastic toxicity literature that commonly reports mass concentrations (μg/mL), we estimated the corresponding mass-based doses for the NPPP-1 exposures.

The NRC Certificate of Analysis for NPPP-1 reports a certified TOC for the NPPP-1 stock dispersion of 300 μg/mL (expanded uncertainty: 27 μg/mL). Assuming that carbon is

predominantly derived from PP (repeat unit C_3H_6), the PP mass concentration of the NPPP-1 stock dispersion is calculated from TOC assuming a carbon mass fraction of 85.7% for PP:

$$C_{PP,stock} = \frac{C_{C,stock}}{f_C} = \frac{300\ \mu g/mL}{0.857} \approx 350\ \mu g/mL$$

Propagation of the expanded uncertainty associated with the certified carbon concentration yields an estimated PP mass uncertainty of:

$$U_{PP,stock} = \frac{27\ \mu g/mL}{0.857} \approx 32\ \mu g/mL$$

The exposure concentration of 1×10^{10} and 1×10^{11} particles/mL of NPPP-1 in this study is calculated from the dilution factor of the stock dispersion.

Supplementary Table S1. Estimated mass-equivalent concentrations for NPPP-1 exposure conditions

Particle number concentration (particles/mL)	Estimated mass concentration (µg/mL)
1×10^{10}	1.0±0.1
1×10^{11}	9.6±0.9

Estimation of mass-equivalent concentrations for SynPP and SynPP-UVd16

Mass-equivalent concentrations for SynPP and SynPP-UVd16 at 1×10^{11} particles/mL were estimated by converting NTA size-resolved number distributions to mass, assuming spherical geometry and a PP density of 0.90 g/cm^3, summing contributions across size bins, and scaling to

the target particle number concentration. Using this approach, SynPP and SynPP-UVd16 correspond to estimated mass concentrations of approximately ~173 μg/mL and 37.6 μg/mL, respectively. These values represent estimates and are subject to NTA size/counting bias and geometric assumptions.

Supplementary Figures

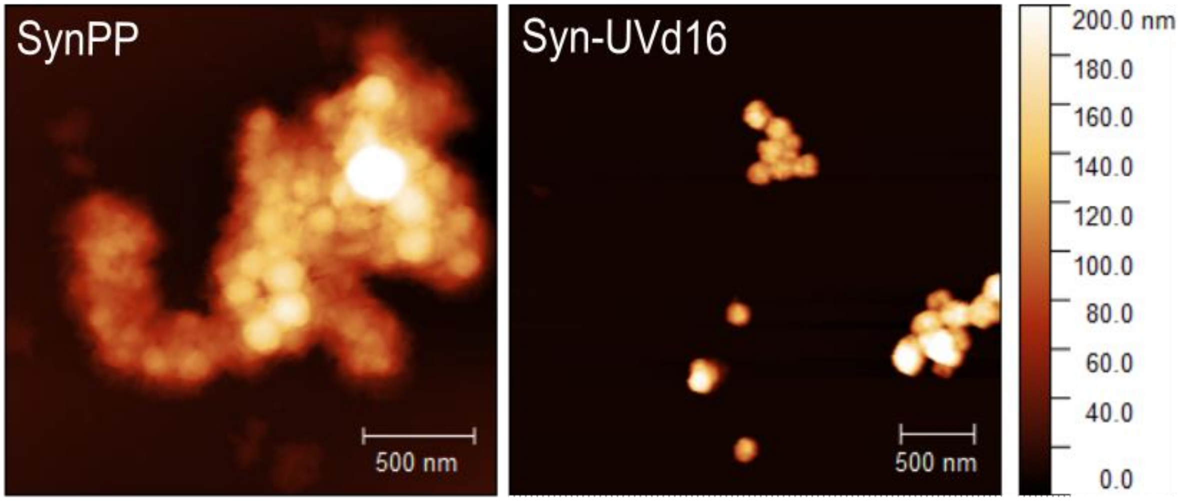

Figure S1. Both SynPP and SynPP-UVd16 particles retain a spherical shape of SynPP. AFM images of SynPP (left panel) and SynPP-UVd16 (right panel).

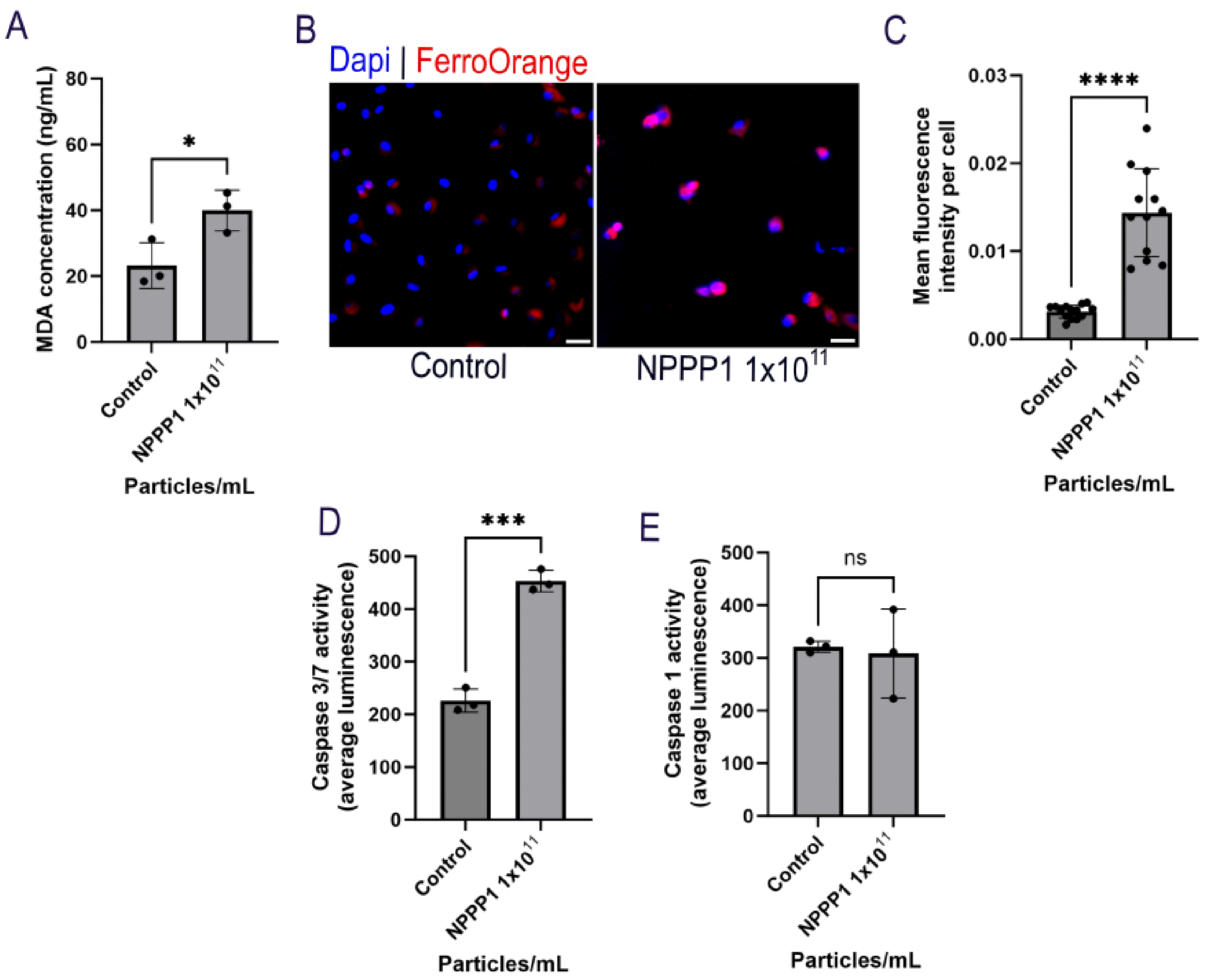

Figure S2. Treatment with NPPP-1 induces ferroptosis and apoptosis in intestinal epithelial cells. (A) Concentration of MDA in the lysates of HIEC-6 cells treated with NPPP-1. (B) Immunofluorescence of FerroOrange (red) showing intracellular Fe^{2+} in HIEC-6 cells treated with NPPP-1. Nuclei is stained in blue (Dapi). Scale bars measure 20 µm. (C) Quantification of intracellular Fe^{2+} determined via measurement of mean fluorescence intensity of FerroOrange on a per cell basis. (D) Apoptosis measured via caspase 3/7 activity in HIEC-6 cells treated with NPPP-1. (E) Pyroptosis measured via Caspase 1 activity in HIEC-6 cells treated with NPPP-1. Values on each graph are shown as mean ± SD of three independent experiments each with replicates (n = 3). Statistical significance was determined with t-test (two groups). $P < 0.05$ was considered significant. *$P < 0.05$; **$P < 0.01$; ***$P < 0.001$; ****$P < 0.0001$. $P > 0.05$, ns: not significant.

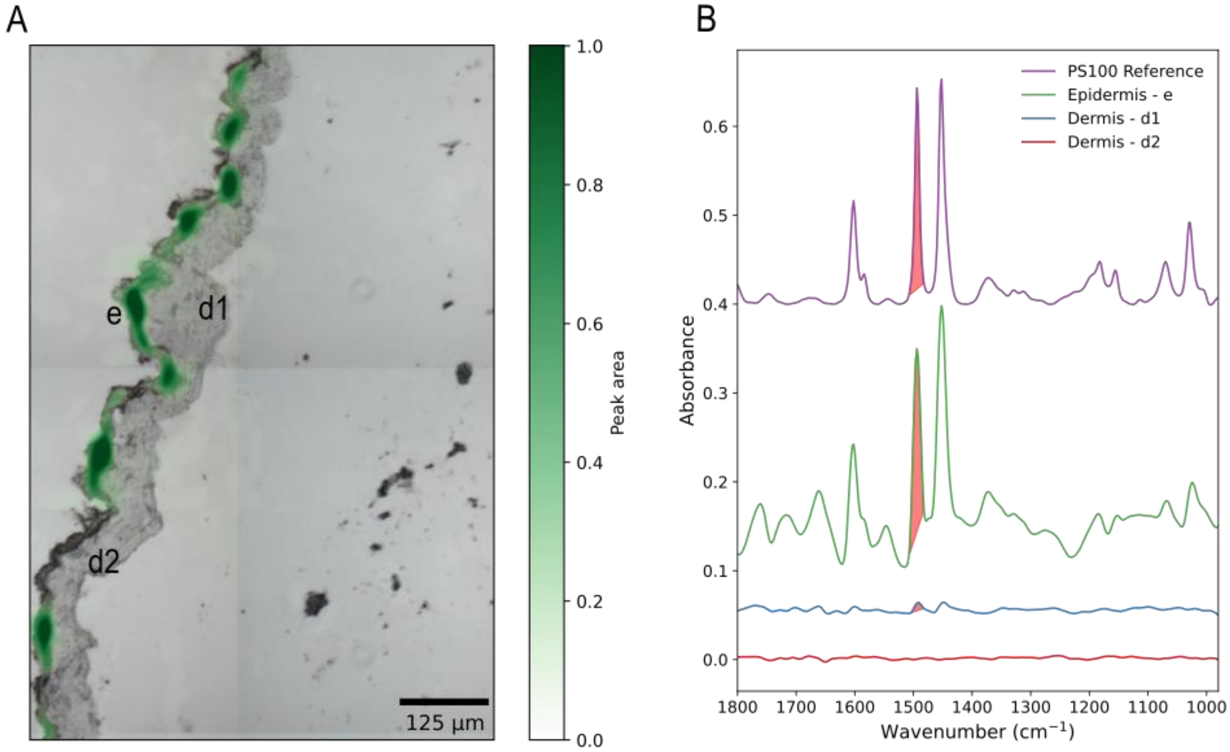

Figure S3. ILIM can be employed to detect the presence of polymers in 3D skin model. (A) µ-IR image highlighting presence of PS in the epidermis (e) and dermis (d1,d2) of in vitro skin model. Image is created by removing the IR signal of the biological background using an autoencoder, and then integrating the peak at 1490 cm^{-1}, as shown in the infrared spectra in (B)

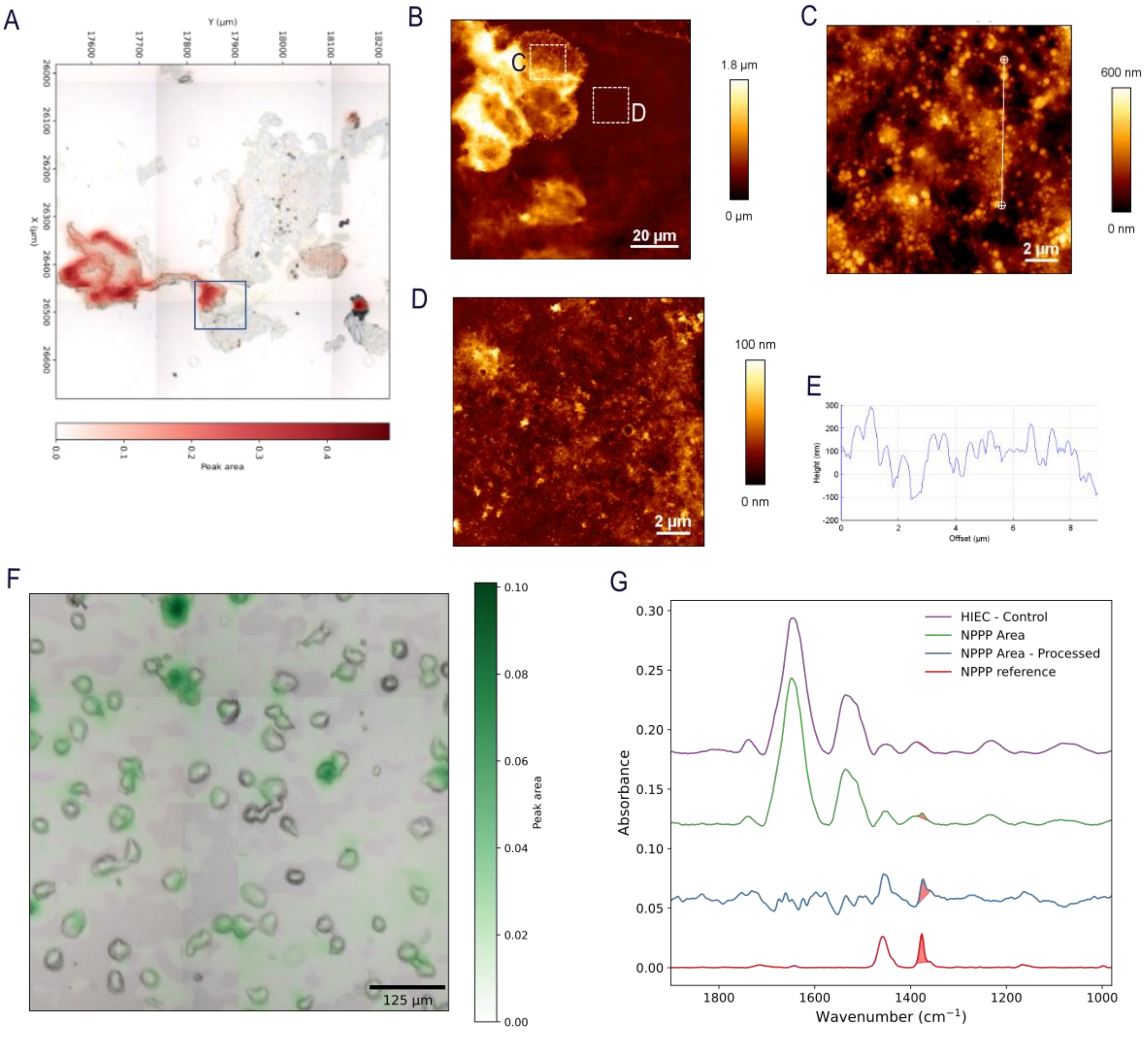

Figure S4. µ-IR and AFM microscopy revealed uptake of NPPP-1 in HSIOs and HIEC-6 cells. (A) Heatmap from µ-IR imaging highlighting regions of PP signal (red). (B-E) Validation of PP uptake by AFM in the area denoted in image A. (C) regions enriched in PP and (D) Regions without PP at IR-reflective slide. (E) Cross section along the selected line in white in image C. (F) A small number of HIEC-6 cells exhibit hotspots showing areas of NPPP uptake. (G) This is observable by IR absorption at 1376 cm^{-1}. Processed spectra (scaled 2×for clarity) show a signature consistent with the original NPPP-1 spectra.
